# Improvement of Sayre’s Treatment for Spinal Curvature

**Published:** 1880-09

**Authors:** 


### IMPROVEMENT OF SAYRE'S TREATMENT FOR SPINAL CURVATURE.

---

Mr. Richard Davy, of London, believes he has an improvement on Dr. Sayre's method of tripod suspension in applying the plaster of Paris jacket in spinal caries. He places the patient in a hammock face downward, arms hanging through slits in the canvass. Extension may then be used or not, according to the views of the surgeon, and the plaster of Paris or other dressing leisurely applied, including the canvas. A free circulation of air is allowed access to the body and the dressing dries rapidly, the patient often sleeping during the time employed. After the drying is complete the spare canvass is trimmed and the patient literally takes up his bed and walks. After reviewing some of the other methods of treating spinal caries according to Sayre's plan, that is of providing an outside support of the body, relieving the weak spinal column, Mr. Davy concludes in favor of his own plan. Aside from the small expense and inconvenience involved, a hammock some times being extemporized from a stout sheet or long night-gown, he thinks suspension not always quite safe in spinal and especially cervical caries, and that in removing the dressing after it causes pain, it is removed too late to avoid mischief. He quotes liberally from the late John Hilton, "On Rest and Pain," and asks if any surgeon after thoughtfully reading Lecture 5, can be ready to fearlessly suspend cases of cervical caries.—*Foreign Cor. American Practitioner.*

